# Hospitalization in Psychiatry: Patients’ experiences at Arrazi Psychiatric Hospital in Morocco

**DOI:** 10.1192/j.eurpsy.2022.1533

**Published:** 2022-09-01

**Authors:** A. Chaara, F. Laboudi, A. Ouanass

**Affiliations:** 1 Arrazi Psychiatric Hospital, Emergency Service, Salé, Morocco; 2 university hospital center, University Psychiatric Hospital Ar-razi, SALE, Morocco; 3 University Hospital Center Ibn Sina, Ar-razi Psychiatric Hospital, Salé, Morocco

**Keywords:** psychiatry, Ethical perspectives

## Abstract

**Introduction:**

Hospitalization in psychiatry is marked by the use of care without consent.

We therefore proposed to study from this perspective the feelings and opinions of patients on such an experience.

**Objectives:**

The objective of this work is to explore the experiences of patients and their perception of the effects of this hospitalization, through 3 fundamental ethical principles: Autonomy, beneficence and non-maleficence.

**Methods:**

This study will be conducted at Arrazi Psychitaric Hospital, in patients at the end of their stay, via a questionnaire.

**Results:**

A total of 122 patients attended the study. A very large proportion of patients were satisfied with the premises, space planning, and had knowledge of a structured planning of the organization of care. A senior doctor was identified by 95% of them. Eighty five per cent were free to move around in the hospital.

The information on the care offered was perceived by 83.7%. The rates are lower with respect to clear explanations received on the disease, the effects of drugs and the type of hospitalization.

Regarding the feelings experienced during the stay, 83% of people who spoke mentioned a painful experience. The feelings that prevailed were a feeling of helplessness, fear, worthlessness. On the other hand, a majority of patients expressed that the hospitalization had protective effects towards themselves and towards others, but that it wasn’t justified.

**Conclusions:**

These results suggest that autonomy and beneficence are respected. Therefore, an attention should be paid to various information given during the stay.

**Disclosure:**

No significant relationships.

